# Ovarian Cancer Radiosensitivity: What Have We Understood So Far?

**DOI:** 10.3390/life13010006

**Published:** 2022-12-20

**Authors:** Amelia Barcellini, Alexandra Charalampopoulou, Loris De Cecco, Andrei Fodor, Emanuela Rabaiotti, Giorgio Candotti, Simona Secondino, Angelica Facoetti, Laura Deborah Locati, Sandro Pignata, Ester Orlandi, Giorgia Mangili

**Affiliations:** 1 Radiation Oncology Unit, Clinical Department, National Center for Oncological Hadrontherapy (CNAO), 27100 Pavia, Italy; 2 Department of Internal Medicine and Medical Therapy, University of Pavia, 27100 Pavia, Italy; 3 Radiobiology Unit, Research and Development Department, National Center for Oncological Hadrontherapy (CNAO), 27100 Pavia, Italy; 4 Molecular Mechanisms Unit, Department of Research, Fondazione IRCCS Istituto Nazionale Dei Tumori, 20133 Milan, Italy; 5 Department of Radiation Oncology, IRCCS San Raffaele Scientific Institute, 20132 Milan, Italy; 6 Unit of Gynaecology and Obstetrics, IRCCS San Raffaele Scientific Institute, 20132 Milan, Italy; 7 Medical Oncology Unit, IRCCS Policlinico San Matteo, 27100 Pavia, Italy; 8 Translational Oncology Unit, Maugeri Clinical Research Institutes IRCCS, 27100 Pavia, Italy; 9 Department of Urology and Gynecology, Istituto Nazionale Tumori, IRCCS-Fondazione G. Pascale Napoli, 80131 Naples, Italy

**Keywords:** ovarian cancer, radiobiology, radioresistance, radiosensitivity, radiation

## Abstract

Radiotherapy has been increasingly considered as an active treatment to combine with other approaches (i.e., surgery, chemotherapy, and novel target-based drugs) in ovarian cancers to palliate symptoms and/or to prolong chemotherapy-free intervals. This narrative review aimed to summarize the current knowledge of the radiosensitivity/radioresistance of ovarian cancer which remains the most lethal gynecological cancer worldwide. Indeed, considering the high rate of recurrence in and out of the radiotherapy fields, in the era of patient-tailored oncology, elucidating the mechanisms of radiosensitivity and identifying potential radioresistance biomarkers could be crucial in guiding clinical decision-making.

## 1. Background

Nowadays, adjuvant whole abdominal radiotherapy (WART), commonly delivered in the past years for ovarian tumors, is no longer considered standard practice due to the high rate of toxicity (gastrointestinal, hematological, and bone) [1]; radical cytoreductive surgery and chemotherapy are the cornerstones of the treatment. WART showed good results in terms of a lower risk of failures [2] and also for the most uncommon histologies such as ovarian clear cell carcinoma [3], which is characterized by unique genotypic and phenotypic signatures and platinum resistance. Although resistance is usually crossed between chemotherapy and radiotherapy (RT), this seems not to be the case for clear-cell ovarian cancers. In particular, the response to WART in the clear-cell histotype seemed to be related to the over-expression of hepatocyte-nuclear-factor 1beta (HNF-1β), which was known as a major culprit to platinum resistance [4]. The WART toxicities were also enhanced when a full dose of chemotherapy was administered in a concomitant or sequential approach [5]. Conversely, the Gynecology Oncology Group described a safe profile of twice-weekly low-dose WART (0.6 Gy in two fractions daily on days 1 and 4 of each week for 6 weeks) delivered as a chemosensitizer for dose-escalated, weekly docetaxel in the recurrent epithelial ovarian fallopian tube, or peritoneal cancers [6]. De Meerleer et al. [7] described the palliative role of WART up to a total dose of 33 Gy over 22 fractions delivered with a modern RT technique, known as intensity-modulated RT (IMRT), in ovarian cancer patients with progressive peritoneal disease. The authors reported a complete response in terms of pain, vaginal bleeding, and bowel obstruction, as well as a reduction of 50% of the therapeutic paracentesis without late gastrointestinal or genitourinary toxicity.

In recent years, RT has played an emerging role in salvage/palliative settings as well as in the prolongation of the chemotherapy-free interval [1,8,9] in oligorecurrent and oligometastatic disease. In particular, thanks to the most recent RT techniques, that allow the delivery of high and ablative doses, RT has lately been increasingly considered as an active treatment to combine with other approaches (i.e., surgery, chemotherapy, and novel target-based drugs) to palliate symptoms and/or to prolong chemotherapy-free-interval [1,8,9]. The clinical largest cohort (449 analyzed lesions) of stereotactic body RT (SBRT) for oligometastatic ovarian cancer described a high profile of efficacy and safety for this approach with a complete response rate of 65.2% and a 24-month actuarial local control rate of 81.9% [9]. Intriguingly, the complete response was associated with a total biologically effective dose α/β10 > 70 Gy, consistent with other solid tumors [9,10,11]. Additionally, other small series confirmed excellent local control rates with mild toxicity [8,12], providing also evidence that endometrioid and clear-cell histology are radiosensitive [13], but the latter had more probability to have a longer chemotherapy-free interval after RT [14]. 

Despite the promising results in terms of local control after SBRT, there was a relevant rate of progression out of the RT fields (from 79 to 100%) [1] and a quota of women that did not achieve a significant response in the RT field. For this reason, in the era of patient-tailored oncology, elucidating the mechanisms of radiosensitivity as well as identifying certain radioresistance biomarkers could be crucial in guiding clinical decision-making [15]. 

Biologically, radiation sensitivity is defined by the survival fraction after 2 Gy (SF2) of radiation, and more than thirty years ago, Slotman and co-workers [16] analyzed the radiation sensitivity of four ovarian cancer cell lines (OVC NOVA, OV 166, OV1225, and OV B09) in vitro observing that OV 166 and OV 1225 were highly radiosensitive (mean inactivation dose 0.82–0.98 Gy, SF2 ≤ 0.13), while OV NOVA and OV B09 had a lower RT sensitivity (mean inactivation dose of 1.31 Gy and SF2 0.22; mean inactivation dose of 1.94 Gy and SF2 = 0.38, respectively) and in addition, the OV B09 cell line had a population doubling time of about four times longer than the other three cell lines. After this pioneering work, the evaluation of radiation sensitivity was implemented in other cell lines and associated with biological features such as BRCA2, TGFbeta-RII, KRAS2, TP53, and/or CDNK2A [17].

Torres-Loca et al. [18] first created a linear regression algorithm to associate the molecular features (i.e., gene expression profiles) and the cellular radiosensitivity over 35 tumor-cell lines recognizing four genes (topoisomerase 1, rbapa48, rgs19, r5pia) strongly related to radiosensitivity. The same research group developed a rank-based linear regression algorithm to predict radiosensitivity. The algorithm is based on a gene-expression signature of 10 genes (radiosensitivity index, RSI) that was directly proportional to cancer radioresistance (high RSI = radioresistance) [19,20]. Eventually, the RSI was adjusted with RT dosimetric parameters, and a genome-based model for adjusting the RT dose (GARD) model was developed [21].

Radioresistance is defined as the ability of cells to overcome the harmful DNA damage caused by RT and, thus, to survive. Several mechanisms are involved in radioresistance (i.e., the anomalous expression of key molecules and genes, the alteration of the tumor microenvironment –TME-, the high hypoxic quota within the tumor, the cold immunophenotype of cancer) that can be intrinsic or acquired [22]. Both kinds of radioresistance can co-exist but while intrinsic radioresistance is due to the dysregulation of molecular pathways that persistently turned on proliferative, invasive, and anti-apoptotic signals, acquired radioresistance is characterized by the alteration of the TME, the dysregulation of the cancer stem cells (CSCs), the enhancement of the DNA repair ability, and autophagic cell death [23,23,24,25,26]. Hypoxia is strongly associated with radioresistance, and several different strategies have been sought to overcome it such as the use of radiosensitizers, targeting the hypoxia-inducible factor as well as delivering high-linear energy transfer (LET) RT. Recently, Wang et al. [27] classified ovarian cancer into two subgroups according to intrinsic hypoxia by a consensus clustering analysis. The “hypoxia 1” group was characterized by a high quota of genetic alterations such as p53 which was higher compared to the “hypoxia 2” group, which showed poor outcomes. In addition, primary ovarian high-grade serous carcinomas were classified into four molecular subtypes (immunoreactive, proliferative, differentiated, and mesenchymal) showing a different clinical outcome with the mesenchymal subtype having the worst prognosis [28]. As a matter of fact, hypoxia has been established as a major promoter of the mesenchymal subtype, promoting adverse treatment outcomes, such as chemo- and radioresistance [29].

**Figure 1:**
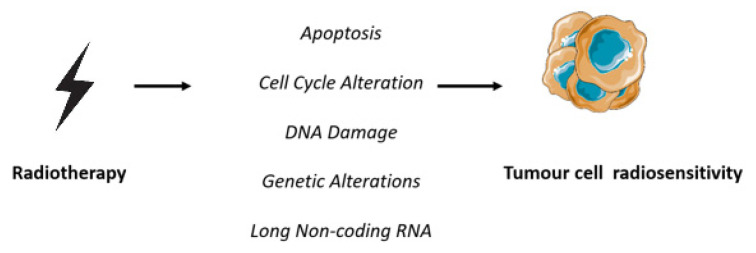
Mechanisms that influence radiosensitivity [30].

The present critical review aims to summarize the current knowledge of the radio-sensitiveness (Figure 1) of ovarian cancer, which remains the most lethal gynecological cancer worldwide and for which RT is being used as an ablative treatment to prolong chemotherapy-free intervals in oligorecurrent and oligometastatic disease.

## 2. Radiation-Induced Apoptosis, Cell Cycle Alteration, and DNA Damage as Predictors of Radiosensitivity

The radiation-induced programmed cell death, known as apoptosis, is the most frequent cell death after radiation exposure. However, the overexpression of anti-apoptotic signals and molecules as well as the dysregulation of pro-apoptotic ones lead to apoptosis escape. These adulterated mechanisms are intensified in radioresistant cellular phenotypes. Hunáková et al. [31] demonstrated the predictive role of radiation-induced apoptosis for radiosensitivity in ovarian cancer cells. They evaluated the radiosensitivity of CH-1, A-2780, P-glycoprotein expressing A-2780/ADR and mismatch repair deficient (MMRd) SKOV-3 with MTT (3-[4,5-dimethylthiazol-2-yl]-2,5-diphenyltetrazolium bromide) assay after exposure to different RT doses (2–10 Gy) divided the cell lines in high radiosensitivity (CH-1; SF2 = 0.60), intermediate radiosensitivity (A-2780/ADR; SF2 = 0.89), and high radioresistance (SKOV-3; SF2 = 0.96). Hence, the authors detected the apoptosis pattern with flow cytometric analysis and found no radiation-induced apoptosis in the SKOV-3 cells, but up to 18% in CH-1 24 h after radiation exposure to 8 Gy. Considering the key role of ADP ribose polymerase (PARP) in recognizing DNA damage, the degradation of PARP was consistently absent in SKOV-3 and present in CH-1, where an 89-kD band in addition to the native 116-kD of PARP was evident on Western blot analysis. These data are of crucial importance considering the pivotal role of PARP inhibitors (PARP-i) in the treatment of ovarian cancer, especially for tumors with DNA repair defects such as BRCA mutation, thanks to their synthetic lethality and the potentiality of combining them to RT in order to overcome radioresistance and effectiveness [32]. Considering the biologic synergy of RT and PARP-i, their potential role as a radiosensitizer has been hypothesized [33]; however, to date, although this interesting strategy seems promising, a better understanding of the clinical implications of ovarian cancer should be investigated. Moreover, considering the possibility to overcome radioresistance with high LET [34], these findings might pave the way to pre-clinical models and following clinical trials using high-LET RT in radioresistant subtypes of ovarian cancers. In this scenario, Keta et al. demonstrated that, compared to photons, high LET was superior in the inactivation of the 59 M ovarian cancer cell line and produced a significant increase in the G2 cell population, as well as a reduction in the S phase [35].

**Figure 2:**
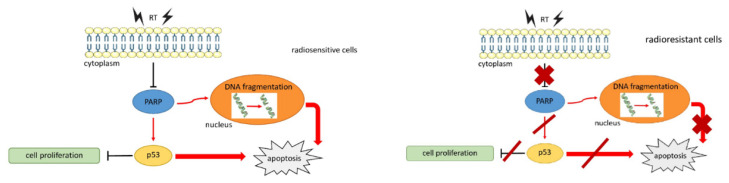
Radiation-induced apoptosis [30].

The use of fractionated schedules in RT finds its rationale in the capability to re-distribute radioresistant cells into more radiosensitive phases in order to achieve better response and to arrest cells at cell cycle checkpoints, especially in the G1, S, and G2 phases, where the crucial radiation-induced cell cycle checkpoints are located [36] (Figure 2).

**Figure 3:**
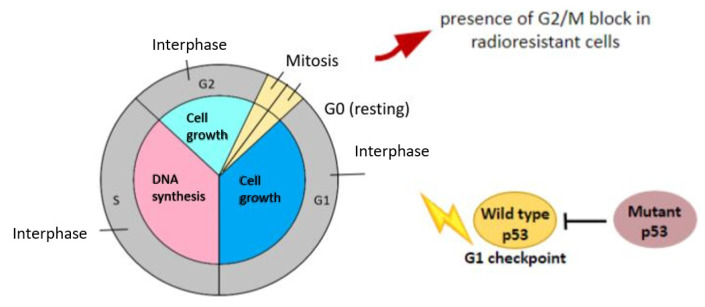
Radiation induces cell cycle alteration and DNA damage [30].

However, cell cycle alterations are frequently observed during RT and can strongly influence radiosensitivity (Figure 3). Considering that p53 controls the G1 checkpoint and p53 is often mutated in cancer cells, tumor cells can take advantage of the S and G2 checkpoints to repair RT damage, and for this reason, combining radiosensitizer drugs with RT can cancel the G2 checkpoint [36]. Hunáková et al. [31], in the work reported above, found that the proportion of cells stopped in G2/M after the RT was higher in the radiosensitive CH-1 (74.5% after 8 Gy) than in the radioresistant SKOV-3 (53.3% after 8 Gy), probably because of their different molecular genetic hallmarks. It is noteworthy that while CH-1 was characterized by a low constitutive level of p53 [37], SKOV-3 was MMRd (large deletion in hMLH1) and p53 was mutated. Moreover, the authors described an absence of a radiation-induced G2/M block after 2 Gy for SKOV-3, but a clear and immediate arrest 24 h after the same dose for CH-1. 

Associated with cell cycle dysregulation, cellular senescence is an important process in ovarian cancer. DNA damage, oxidative stress, and oncogene stress induce a phenotype of cellular senescence. Cellular senescence might also be induced by radiation, altering X-ray sensitivity in tumor cells [38,39]. Senescent cells show apoptosis resistance and are able to produce proinflammatory and profibrotic molecules, causing sensible changes in the microenvironment; on the one hand, the radio-induced tumor senescent cell can regrow, and on the other hand, the normal senescent cell may induce fibrosis or promote tumorigenesis, while non-senescent cells may become senescent or resistant to radiation [40]. The molecular mechanisms of cellular senescence induced by X-ray irradiation were investigated, taking into account the network hub played by the activating transcription factor 7 (ATF7), a stress-responsive recruiter of histone H3K9 di- and trimethyltransferases. ATF7 acts by silencing CDKN2B and recruiting G9a histone H3K9 dimethyltransferase, while X-ray irradiation cancels this epigenetic silencing via ATF7 phosphorylation [41]. In addition, a pre-clinical study demonstrated the induction of a cell line-dependent senescence-like phenotype after a combination approach with RT (photons and protons) and noncytotoxic concentration of PARP-i. Therefore, senescence-targeting inhibitors could enhance the effects of combining PARP-i with conventional or proton RT [42]. Taken together, a better understanding of cell fates following RT is expected to improve the therapeutic modalities to enhance, eventually, cell death and reduce adverse effects.

**Figure 4:**
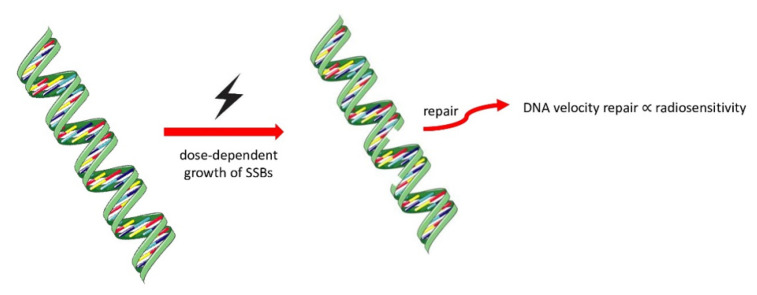
The rapidity of DNA repair was inversely proportional to radiosensitivity (CH-1 < A-2780 < SKOV-3) [30].

Bacová et al. [43] evaluated the post-actinic DNA damage in CH-1, A-2780, and SKOV-3, finding a dose-dependent growth of DNA single-strand breaks (SSBs), with a statistically significant difference in the levels between CH-1 (higher levels), SKOV-3 (lower levels), and A-2780 (intermediate levels) reflecting the intrinsic different levels of radiosensitivity (Figure 3). The authors found that the rapidity of DNA repair was inversely proportional to radiosensitivity; indeed CH-1 was the slowest, and A-2780 and SKOV-3 were the most rapid. Despite the similarity in DNA repair speed, the types of DNA damage in A-2780 after 2 and 8 Gy were more similar to CH-1 and not to SKOV-3 (Figure 4). The higher level of radioresistance of SKOV-3 was also reported by Petru et al. [44] using the ATP cell assay. 

## 3. Genetic Alterations and Non-Coding RNA as Predictors of Radiosensitivity

**Figure 5:**
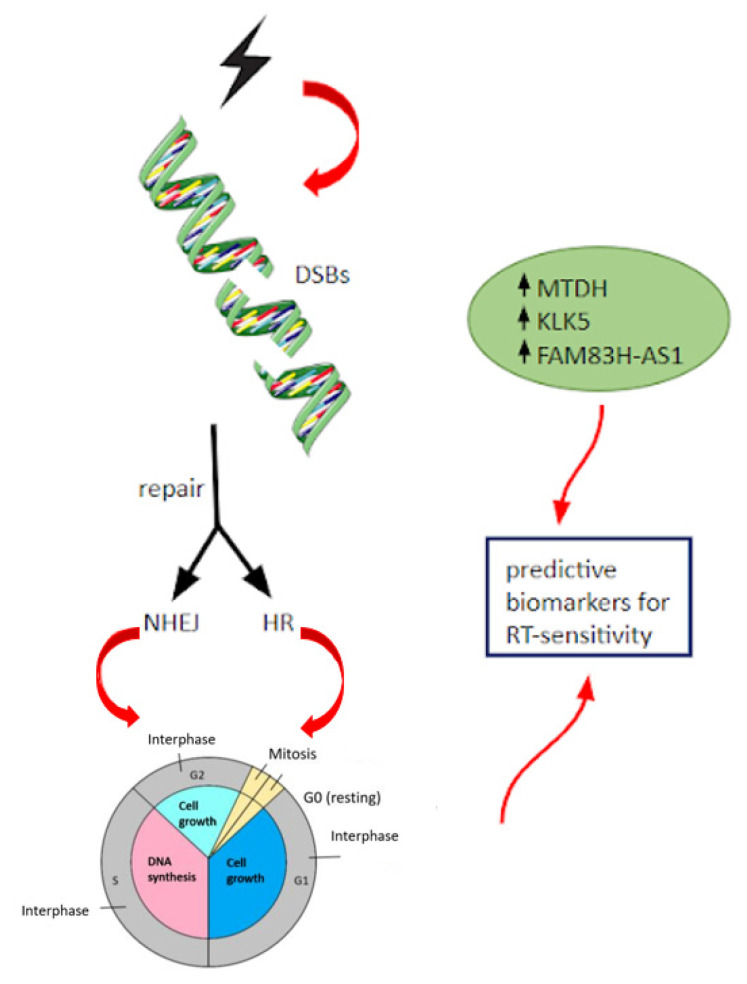
Radio-induced DNA damage and non-coding RNA as a predictor of radioresistance [30].

Molecular and genetic alterations involving DNA repair have proven to be related to a different response to RT, and in particular, defects in DNA damage repair are correlated to radiosensitivity and radiosusceptibility (Figure 5) [45]. The most common radio-induced DNA damage (double-strand breaks-DSBs) can be repaired through a non-homologous end-joining (NHEJ) or homologous recombination (HR) pathway based on the cell cycle phases (NHEJ in G1, HR in mid-S and mid-G2 phases) [46]. BRCA1 and BRCA2 play a pivotal role in HR, and a higher risk of contralateral breast cancer was described in patients with germline mutation who underwent adjuvant RT after breast surgery [47]. Kim et al. [48] described a significantly higher objective response and a lower local recurrence rate in patients with BRCA mutation treated with RT and a dramatic and durable response when there was also a concomitant ATM mutation (1-year local recurrence rate at 0% in BRCA + ATM mutated vs. 47.9% in wild type, *p* = 0.008) without an increased toxicity rate.

Mutation in tumor suppressor gene p53, which is involved in cell-cycle control, DNA repair, and apoptosis, is the most prevalent mutation in ovarian cancer and is correlated with the resistance to chemotherapy and RT due to the annulment of p53-dependent apoptosis. Concin et al. [49] probed the accumulation after RT as well as Bcl-2 expression before and after RT in three lines of ovarian carcinoma cells (PA-1, Caov-3, and SKOV-3), finding that p-53 mutant cell lines (SKOV-3 and Caov-3) were radioresistant at different RT doses with no p-53 accumulation after RT compared to PA-1, which was a p-53 wild type and showed an accumulation of p53 after radiation exposure. Moreover, the authors found that radio-responsiveness dependent on p-53 status was associated with the growth rate (lower for Caov-3 and SKOV-3 and higher for PA-1). Langland et al. [50] analyzed 16 ovarian cancer cell lines and probed that those radioresistant were mostly p-53-mutated, confirming that p-53 status may be considered a predictive biomarker for radiosensitivity. Moreover, the authors [50] measured the levels and activity of DNA-dependent protein kinases (DNA-PK) that play a decisive role in the repair of the post-actinic DSBs and showed significant variation between different cell lines without correlation with radiosensitivity profiles suggesting that they play no role in the prediction of radiosensitivity in ovarian cancer. In recent years, metadherin (MTDH), an oncogene implicated in cancerogenesis [51], has been recognized as a remarkable prognostic marker of several malignancies such as ovarian cancer. It was demonstrated [52] that silencing MTDH in SKOV-3 cells significantly increased their radiosensitivity, post-actinic apoptosis, and tumor growth after RT. 

Kallikrein-related peptidase 5 (KLK5) is implicated in tumor progression, and its expression seems to impact the prognosis of several solid tumors [53,54,55] such as ovarian cancers. A strong negative correlation between the KLK5 mRNA levels and the prognosis was found in advanced high-grade serous ovarian cancer [56]. Even if its overexpression has been reported in radioresistant cancer tissues [57], its impact on ovarian radiosensitivity is still unknown. 

Long non-coding RNA (lncRNA) is a class of transcripts over 200 bases with no protein-coding ability that participate in several tumor processes including proliferation, metastasis, and responsiveness to oncological treatments. Dou et al. [58] demonstrated that the upregulation of lncRNA FAM83H antisense RNA1 (FAM83H-AS1) was related to radioresistance through Hu-Antigen R (HuR), an RNA-binding protein, and its knockdown promoted radiosensitivity, suggesting its predictive role in radio-responsiveness. 

MicroRNAs are small, single-stranded, non-coding RNAs acting as post-transcriptional regulators of gene expression. Zhao et al. [59] identified miR-210 as being able to promote the proliferation, migration, and invasion of ovarian cancer cells. The process involves the activation of the AKT signaling pathway and Epithelial-mesenchymal transition-related genes resulting in a reduction in the sensitivity of ovarian cancer cells to RT.

## 4. Clinical Implication and Future Perspectives

Due to the potential tumor chronicity promoted by the administration of anti-VEGF [60] and/or PARP-i [61] in oligo-recurrent and oligo-metastatic ovarian cancers, the clinical management of these lesions is becoming increasingly challenging. In this clinical context, RT moves from a palliative to a curative aim in a multimodal approach that integrates hypofractionated schedules with surgery, chemotherapy, and new target-based drugs.

The challenge is understanding which patients might benefit most from RT alone or in combination with other therapies. Elucidating the mechanisms of radiosensitivity, and finding certain biomarkers of radioresistance might be crucial in guiding clinical decision-making [15]. Bi et al. [62] analyzed in vitro and in vivo the radiosensitizing capability of Olaparib on BRCA1-proficient and BRCA1-deficient high-grade serous ovarian carcinoma cell lines, showing that the response to RT alone was worse for BRCA1-proficient cells in which there were also lower Olaparib-mediated radiosensitization effects. Moreover, as opposed to BRCA1-proficient tumors, mice bearing BRCA1-deficient tumors showed more pronounced growth inhibition with Olaparib and RT treatment. The question might be how to increase the effectiveness of RT with or without Olaparib in radioresistant cells without worsening the toxicity profiles [32].

Radioresistance can be overcome using different strategies, such as hypofractionaction, that are typical of SRBT schedules or applying radiation with higher relative biological effectiveness (RBE) and linear energy transfer (LET), which are typical characteristics of particle beam RT. In this scenario, carbon ions appear really promising and effective against hypoxic tumors also for their intrinsic independence on the tissue oxygenation status [34]. Understanding the mechanisms of radio-responsiveness might improve the development of a rapid assay to predict the RT response as well as to select patients to enroll in more aggressive treatment. 

No less, preclinical and clinical evidence showed the pro-immunogenic role of RT and the possibility to harness radiation to improve the efficacy of immunotherapy [63,64,65,66,67] also with low doses of RT [68]. It has been proven through patient tumor-derived spheroids that ovarian cancers are responsive to a low dose of radiation, with a mean inactivation dose that ranges between 1.31 Gy and 2.80 Gy [69]. Low-dose WART (0.6 Gy in two daily fractions on day 1 and day 5 of each week for 3 weeks) was tested also in a combination approach with Veliparib for epithelial ovarian, fallopian tube, or peritoneal cancers, achieving an objective response in platinum-sensitive BRCA-mutated ovarian cancer and an overall survival in the platinum-sensitive population of 11 months and in platinum-resistant patients of 6 months [70]. Low-dose RT has proved to be worthwhile in reversing immune desertification and resistance to immunotherapy [71]. In this context, Herrera et al. [71] worked on a phase I trial aimed at reprogramming the TME of immunological desert tumor phenotypes ( including high-grade serous ovarian cancers) into hot ones through the combination of low-dose RT and low-dose cyclophosphamide and immune checkpoint blockade. The authors found that the combo-approach triggered adaptive and innate immunity and raised mostly CD4+ T cells with Th1 signatures to infiltrate TME, reverting the immunological phenotypes and inducing dramatic responses in metastatic immune-cold tumors. Indeed, the response to immunotherapy depends on the presence of tumor-specific T cells in the TME.

In this new and fascinating scenario, it can be intriguing to test the effect of high-LET RT in order to evaluate its potential and additional advantages. The dosimetric characteristics of high-LET RT allow to spare better the normal tissues such as the bone marrow as well as the circulating lymphocytes compared to photon beam RT. It is widely acknowledged that lymphopenia is less pronounced in patients treated with proton beam RT and carbon ion RT compared to patients treated for the same volumes and disease with conventional RT [72,73], and this is certainly an advantage for ovarian cancer patients who underwent often several lines of chemotherapy. Moreover, due to the capability of reducing the volumes of surrounding normal tissues irradiated, a significantly lower number of chromosomal aberrations were recorded in patients treated with carbon ions than with photons [74]. For this reason, carbon ion radiotherapy (CIRT) patients are less lymphopenic with a higher number of lymphocytes without chromosomic alterations that might support a more effective immune response. In addition, the dosimetric advantage of CIRT allows to spare lymph nodes, and this might be crucial considering the emerging data suggesting that elective nodal irradiation may decrease immune response [75].

In advanced ovarian cancers, many immune suppressive factors might weaken the T effector cells, negatively impacting the response to immunotherapy and in particular to PD-1 inhibitors [68]. In a monocentric, open-label, phase II trial, twenty women with advanced or recurrent, platinum-resistant ovarian cancer were treated with anti-PD1 agents (Nivolumab at the dose of 1–3 mg/kg), and even if there was no significant association between the expression of PD-L1 and the objective response, 12.5% (2 of 16 patients) of cases in which was recorded high PD-L1 expressions experienced an objective response compared to the absence of response in 2 of the 4 patients with a low expression of PD-L1. The question is how to increase PD-L1 expression in ovarian cancers [76]. In irradiated cancer cells, Sato et al. [77] demonstrated an up-regulation of the PD-L1 expression in response to DSBs, through the ATM/ATR/Chk1 kinase pathway. This effect was found also after CIRT and seemed to be dose-dependent [78]. Indeed, Iijima et al. [78] found that 44% (8 of 18 women) of cervical adenocarcinoma patients that had no PD-L1 expression before treatment, experienced an up-regulation after CIRT that significantly impacted their progression-free survival supporting the combination of immune checkpoint inhibitors and high-LET RT. Radiation-damaged tumor cells release various immunological mediators (i.e., ATP, high-mobility group box 1, calreticulin), enhancing the concentration of effector T cells in the microenvironment [74]. Moreover, DNA DSBs in the cell’s cytosol activate the interferon-type-1 (IFN-I) via the cGAS/hSTING pathway [79]. In the meantime, the immunological cell death induced by RT promotes the cross-presentation of tumor-derived antigens by dendritic cells to T cells [80]. Together these processes trigger an inflammatory response promoting dendritic cell recruitment and a cross-priming of T effector cells that are a key point to turning tumor cells into an in situ vaccine [63,66,68,81]. A potential option could be using a pro-immunogenic RT such as CIRT to convert so-called cold or immune-desert cancers to hot ones to fuel the immune responses. The combination of immune checkpoint inhibitors and high-dose RT [82] was proven to be safe also with CIRT [83], and preliminary animal studies have proved that high-LET RT effectively provokes abscopal responses on metastasis after the irradiation of the primary tumor [84,85,86]. 

Considering the emerging preclinical and clinical data about the combination of immunotherapy with RT and the pro-immunogenic role of high-LET RT, finding a molecular signature of radiosensitiveness both in immune-competent and immune-desert phenotypes might guide clinicians to build future studies for the combination of RT with immunotherapy in oligorecurrent/oligometastatic ovarian cancers. 

## 5. Conclusions

To the best of our knowledge, there is no report concerning the radio responsiveness of ovarian cancers. Despite the unavoidable limitation of the current review (narrative, small data, lack of data on high-LET RT that might be more effective on radioresistant subtypes), we found that several hallmarks are worthwhile in defining the response of ovarian cancer to RT such as RT-induced apoptosis, cell cycle alteration, DNA damage, senescence, molecular and genetic alterations. Considering the increasing role of RT in the treatment of the oligorecurrence and oligoprogression of ovarian cancer, understanding the mechanism of radio-responsiveness is of great interest. Indeed, in a tailored oncological approach, classifying the patients based on their radio-responsiveness might improve decision-making. Large preclinical registries of ovarian cancer tissues and cells might contribute to the construction of a dedicated predictive radiosensitivity index. Clinical studies testing the combination between RT and PARP-i or immunotherapy should be designed in order to make a step forward in the comprehension of the optimal time window, fractionation schedules, and types of RT able to potentiate the biological and oncological response.

**Figures**: Parts of the figures were drawn by using pictures from Servier Medical Art (smart.servier.com, accessed on 7 November 2022). Servier Medical Art by Servier is licensed under a Creative Commons Attribution 3.0 Unported License (https://creativecommons.org/licenses/by/3.0/, accessed on 7 November 2022).
